# Prognostic Value of DNA Methylation-Driven Genes in Clear Cell Renal Cell Carcinoma: A Study Based on Methylation and Transcriptome Analyses

**DOI:** 10.1155/2020/8817652

**Published:** 2020-07-11

**Authors:** Maolin Hu, Jiangling Xie, Huiming Hou, Ming Liu, Jianye Wang

**Affiliations:** ^1^Peking University Fifth School of Clinical Medicine, Beijing 100730, China; ^2^Department of Urology, Beijing Hospital, National Center of Gerontology, Beijing 100730, China; ^3^Department of Urology, Qianjiang Central Hospital of Chongqing Municipality, Chongqing 409000, China

## Abstract

**Background:**

Few previous studies have comprehensively explored the level of DNA methylation and gene expression in ccRCC. The purpose of this study was to identify the key clear cell renal cell carcinoma- (ccRCC-) related DNA methylation-driven genes (MDG) and to build a prognostic model based on the level of DNA methylation.

**Methods:**

RNA-seq transcriptome data and DNA methylation data were obtained from The Cancer Genome Atlas. Based on the MethylMix algorithm, we obtain ccRCC-related MDG. The univariate and multivariate Cox regression analyses were employed to investigate the correlation between patient overall survival and the methylation level of each MDG. Finally, a prognosis risk score was established based on a linear combination of the regression coefficient derived from the multivariate Cox regression model (*β*) multiplied with the methylation level of the gene.

**Results:**

19 ccRCC-related MDG were identified. Three MDG (NCKAP1L, EVI2A, and BATF) were further screened and integrated into a prognostic risk score model, risk score = (3.710∗methylation level of NCKAP1L) + (−3.892∗methylation level of EVI2A) + (−3.907∗methylation level of BATF). The risk model was independent from conventional clinical characteristics as a prognostic factor for ccRCC (HR = 1.221, 95% confidence interval: 1.063–1.402, and *P* = 0.005). The joint survival analysis showed that the gene expression and methylation levels of the prognostic genes EVI2A and BATF were significantly related with prognosis.

**Conclusion:**

This study provided an important bioinformatics foundation for in-depth studies of ccRCC DNA methylation.

## 1. Introduction

Clear cell renal cell carcinoma (ccRCC) is the most common pathological type of renal cancer [1, 2]. Approximately 350,000 new patients with RCC are confirmed globally every year, and RCC causes more than 140,000 deaths each year [3]. ccRCC has complex biological characteristics and is not sensitive to radiotherapy and chemotherapy; radical or partial nephrectomy is the main method for treating RCC [4, 5]. Although the surgical effect is definite, 20%-40% of patients experience local recurrence or distant metastasis after surgery [6], and the mortality rate is very high for advanced renal cell carcinoma [7]. In the last ten years, with the development of molecular biology, several ccRCC driver genes such as von Hippel–Lindau (VHL) and PBRM1 have been discovered [8, 9], and targeted drugs, such as tyrosine kinase inhibitors (TKIs) [10], have been developed to treat these advanced ccRCC patients; however, not all of these patients can benefit from these drugs. Therefore, the identification of new ccRCC-related driver genes and the establishment of risk models through bioinformatics analysis are very important for patient prognosis evaluation.

Epigenetic changes are thought to be closely related to cancer progression, and aberrant DNA methylation is one of the most crucial and routine epigenetic modifications [11]. DNA methylation is an epigenetic modification that plays a crucial role in regulating the growth, development, gene expression pattern, and stability of the genome without changing the DNA sequence [12, 13]. Many studies in recent years have found that abnormal DNA methylation is closely related to tumorigenesis, development, and cell canceration [14–16]. In particular, alterations in DNA methylation can supply crucial information for the early diagnosis and prognosis of cancer [17–20].

Few previous studies have comprehensively explored the level of DNA methylation and gene expression in ccRCC. The purpose of this study was to identify the key ccRCC-related methylation-driven genes and to build a prognostic model based on the level of DNA methylation.

## 2. Materials and Methods

### 2.1. Data Acquisition and Integrative Analysis

In this study, RNA-seq transcriptome data and DNA methylation data were obtained from The Cancer Genome Atlas (TCGA) website (https://portal.gdc.cancer.gov/repository). Of them, the DNA methylation data was using the Illumina Infinium HumanMethylation450 platform and 27 platform, and beta values, ranged from 0 to 1, were quantified to indicate the levels of DNA methylation. Based on R software and packages [21], we analyzed the above data to obtain differentially methylated genes and differentially expressed genes. In addition, clinicopathological data including survival status, survival time, age, sex, the International Society of Urological Pathology (ISUP) grade, and American Joint Committee on Cancer (AJCC) stage were obtained from TCGA. Based on the MethylMix package [22], we performed an analysis by combining the differentially methylated genes and differentially expressed genes. MethylMix is an algorithm for identifying the hypermethylation and hypomethylation of genes in diseases [23]. MethylMix is based on a *β*-mixture model to identify the methylation status and compares this status with the normal DNA methylation state. The correlation was computed between the gene methylation level and the gene expression level. Finally, the exported result of MethylMix is methylation-driven genes (MDG).

### 2.2. MDG Set Enrichment Analysis

Gene functional enrichment analyses were conducted to discover the main biological characteristics of the MDG, including Gene Ontology (GO) analyses with molecular function, biological process, and cellular component analyses [24]. In our study, the biological process category was selected for GO analysis through the enrichGO function in the clusterProfiler package (version 3.6) and the original database was acquired from the “org.Hs.eg.db” package [25].

### 2.3. Definition of the MDG-Related Prognostic Model

We randomly divided the tumor samples obtained from TCGA into two cohorts: a training cohort (244 patients, Table S1) and a validation cohort (242 patients, Table S2). We used the training cohort to build the Cox regression risk model, and the validation cohort was adopted to verify the performance of the model. First, we performed univariate Cox regression analysis to identify six methylation-driven genes related to overall survival in patients by considering *P* value < 0.01 as significant. Then, multivariate Cox regression analysis was used to build a prognostic risk model. The risk score was figured out using the regression coefficients (*β*) from the multivariate Cox regression model to weight the methylation values of the selected genes. Risk score = *β*gene (1) × methylation level of gene (1) + *β*gene (2) × methylation level of gene (2) + ⋯+*β*gene (*n*) × methylation level of gene (*n*). According to the median risk score, the training cohort was separated into the low-risk and high-risk groups. To assess the predictive power of the risk score, we performed receiver operating characteristic (ROC) curve analysis. The same method was adopted in the testing cohort to test the performance of the model.

### 2.4. Independence of the Risk Model from Traditional Clinical Features and Building a Nomogram

To verify whether the risk score was independent of other clinical variables in ccRCC patients (including age, sex, ISUP grade, and AJCC stage), the univariate and multivariate Cox regression analyses were performed on the entire TCGA cohort. According to results of multivariate Cox regression analysis, we constructed a nomogram with R package “rms” to assess the overall survival for ccRCC. The predictive performance was evaluated by the *C* index and calibration curve.

### 2.5. Joint Survival Analysis of the Gene Expression Levels and Methylation Levels of the MDG

To further identify the key genes related to the prognosis of ccRCC patients, based on the survival R package, a joint survival analysis was performed by combining the methylation levels of MDG with the corresponding gene expression levels.

### 2.6. Statistical Analysis

All statistical analyses were performed using R software. *P* < 0.05 was considered statistically significant. The MethylMix package was used to identify DNA methylation-driven genes. We performed the univariate and multivariate Cox regression analyses to determine whether the risk score had prognostic value independent of various clinicopathological characteristics. We conducted the log-rank test and Kaplan-Meier survival analysis to evaluate the predictive ability of the risk model.

## 3. Results

### 3.1. Data Analysis and Acquisition of DMG in ccRCC

**Figure 1:**
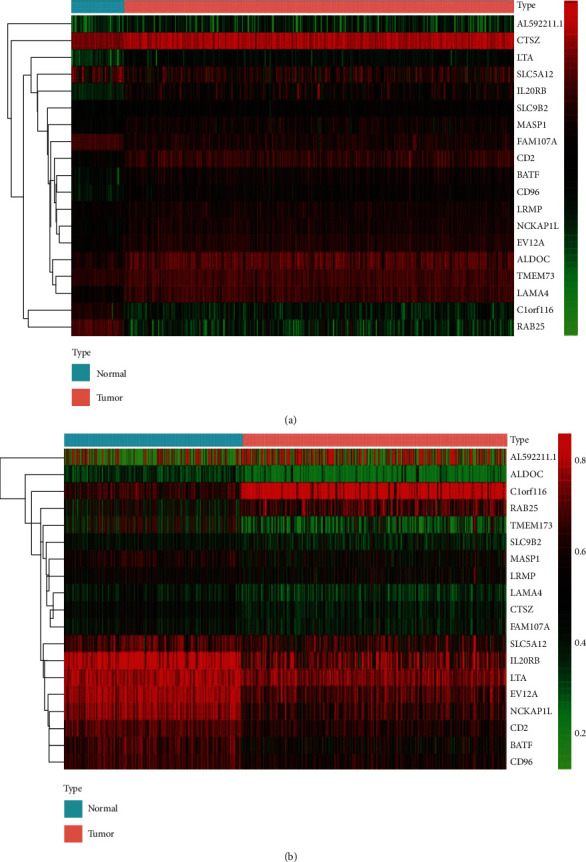
Heat maps of ccRCC-related aberrant methylation-driven genes. (a) The hierarchical clustering heat map of ccRCC-specific methylation-driven mRNAs. (b) The hierarchical clustering heat map of the methylation level of ccRCC-specific methylation-driven genes. In the figure, red represents highly methylated genes and green represents low methylated genes between ccRCC and normal tissues.

All data were acquired from TCGA. The methylation data were downloaded from 542 cancer tissues and 357 noncancer tissues. In addition, the mRNA expression data were retrieved from 611 samples, consisting of 72 normal samples and 539 cancer samples. On the basis of the LIMMA software package, we analyzed the downloaded data to obtain differentially methylated genes and differentially expression genes. We performed an integrative analysis via the R package MethylMix, and the analysis required three datasets, including normal DNA methylation data, cancer DNA methylation data, and matched gene expression data (Tables S3, S4, and S5). ∣logFC | >0, *P* < 0.05, and ∣Cor | >0.3 were adopted for screening MDG. Finally, 19 MDG were obtained (Table 1). Heat maps of the ccRCC-related aberrant MDG are shown in Figure 1.

### 3.2. MDG Set Enrichment Analysis in ccRCC

**Figure 2:**
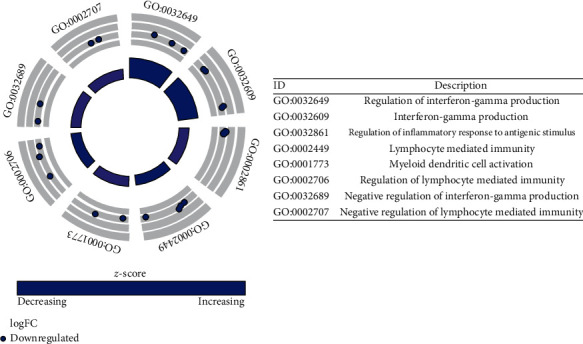
Functional enrichment analysis of methylation-driven mRNAs in ccRCC. The outer circle represents the expression (logFC) of methylation-driven mRNAs in each enriched GO (gene ontology) term: red dots on each GO term indicate upregulated methylation-driven mRNAs and blue dots indicate downregulated methylation-driven mRNAs. The inner circle indicates the significance of GO terms.

Gene Ontology analysis showed that MDG were mainly enriched in the regulation of interferon-gamma production, interferon-gamma production, regulation of the inflammatory response to antigenic stimuli, lymphocyte-mediated immunity, myeloid dendritic cell activation, regulation of lymphocyte-mediated immunity, negative regulation of interferon-gamma production, and negative regulation of lymphocyte-mediated immunity (*P* < 0.05, Figure 2).

Above MDG set enrichment analysis revealed that MDG were significantly linked to inflammatory response regulation and immune regulation.

### 3.3. Construction of the MDG-Related Prognostic Model

**Figure 3:**
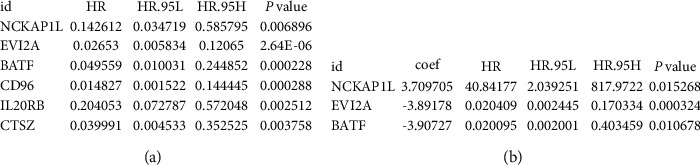
Further analysis of methylation-driven genes the in the training cohort. (a) Six methylation-driven genes selected through univariate Cox regression analysis. (b) Final multivariate prognostic model containing three survival-associated methylation-driven genes.

**Figure 4:**
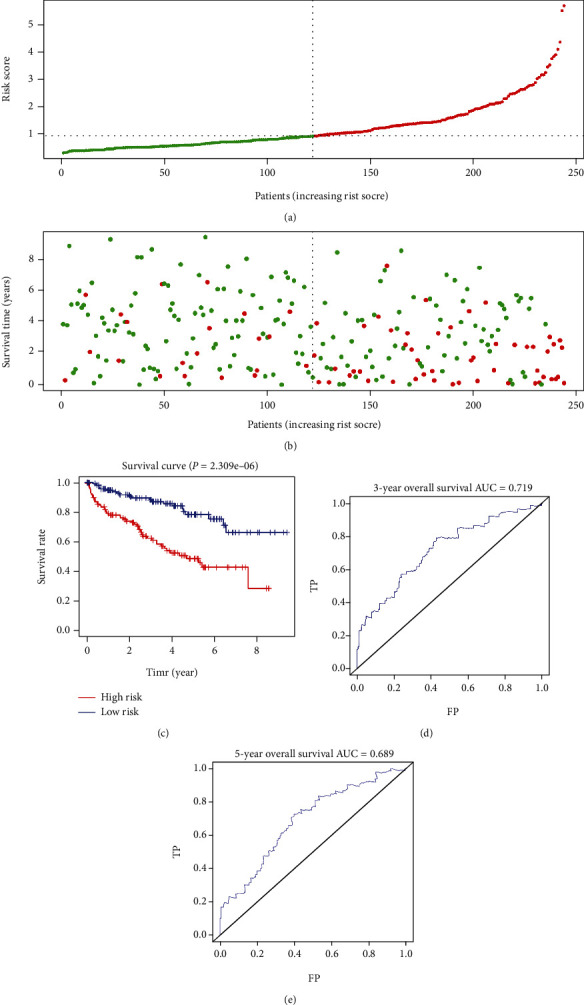
Prognostic analyses of the high-risk and low-risk patients in the training cohort. (a) Risk score distribution of patients in the prognostic model. (b) Survival status scatter plots for patients in the prognostic model (green dots: alive; red dots: death). (c) The Kaplan-Meier plot (high-risk vs. low-risk group) for the training cohort. (d, e) Receiver operating characteristic curves showed the predictive efficiency of the risk score for the training cohort. AUC: area under the curve; FP: false positive; TP: true positive.

The training cohort was used to construct the risk model. Firstly, we conducted a univariate Cox regression analysis of the methylation level of nineteen MDG. We identified six genes (NCKAP1L, EVI2A, BATF, CD96, IL20RB, and CTSZ) related to overall survival in the training cohort by considering *P* value < 0.01 as significant (Figure 3(a), *P* value < 0.01). To better predict the relationships between the methylation levels of the methylation-driven genes and overall survival of ccRCC, we further analyzed them by stepwise multivariate Cox regression analysis. Finally, three genes were selected to build a predictive model (Figure 3(b), *P* value < 0.05). The risk score was figured out using a linear combination of the methylation levels of the three selected MDG weighted by their specific regression coefficients (*β*). Risk score = (3.710∗methylation level of NCKAP1L) + (−3.892∗methylation level of EVI2A) + (−3.907∗methylation level of BATF). On the basis of the median risk score, the patients in the training cohort were classified as a high-risk group and a low-risk group. The risk score distribution of the patients according to the prognostic model is shown in Figure 4(a). Survival status scatter plots for the patients according to the prognostic model are shown in Figure 4(b), which shows that the high-risk subgroup contained a higher number of patients who died than the low-risk subgroup. We observed a significant difference in overall survival between the two groups (Figure 4(c), *P* < 0.0001), and the AUCs at three and five years were 0.719 and 0.689, respectively (Figures 4(d) and 4(e)).

### 3.4. Testing of the Methylation-Driven Gene-Related Prognostic Model

**Figure 5:**
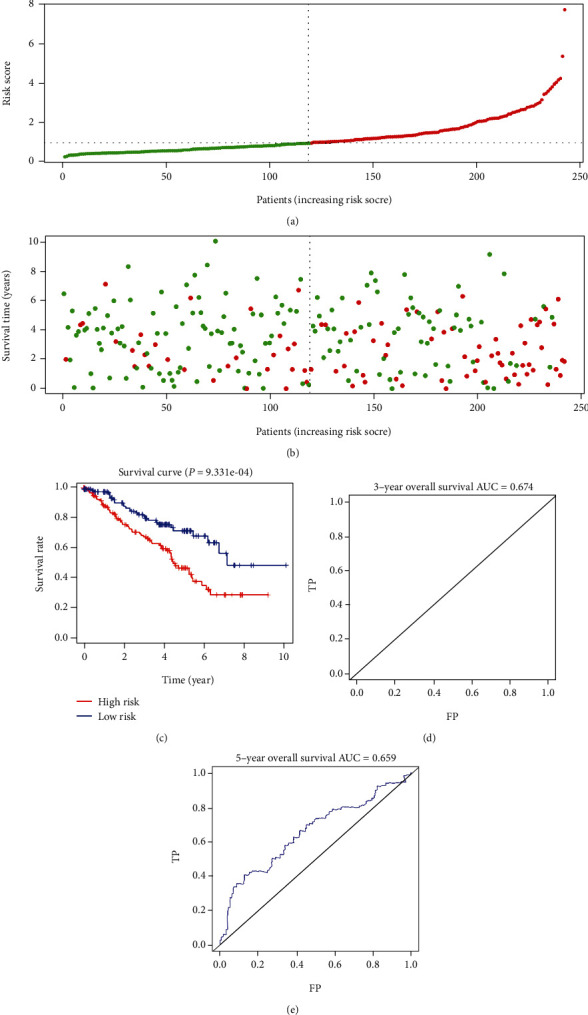
Prognostic analyses of the high-risk and low-risk patients in the testing cohort. (a) Risk score distribution of patients in the prognostic model. (b) Survival status scatter plots for patients in the prognostic model (green dots: alive; red dots: death). (c) The Kaplan-Meier plot (high-risk vs. low-risk group) for the testing cohort. (d, e) Receiver operating characteristic curves showed the predictive efficiency of the risk score for the testing cohort. AUC: area under the curve; FP: false positive; TP: true positive.

To confirm the performance of the model, the testing cohort was analyzed. First, we used the selected methylation-driven genes (NCKAP1L, EVI2A, and BATF) to compute the risk score of each patient in the validation cohort. Similar results were observed in the testing cohort, and the risk score distribution of the patients according to the prognostic model is shown in Figure 5(a). Survival status scatter plots for the patients according to the prognostic model are shown in Figure 5(b). Significant survival differences were observed in the testing cohort (Figure 5(c), *P* < 0.0001). The AUCs at three and five years were 0.674 and 0.659, respectively (Figures 5(d) and 5(e)).

### 3.5. Independence of the Prognostic Model from Other Clinical Characteristics and Building a Nomogram Based on the Risk Score and Other Clinical Characteristics

**Figure 6:**
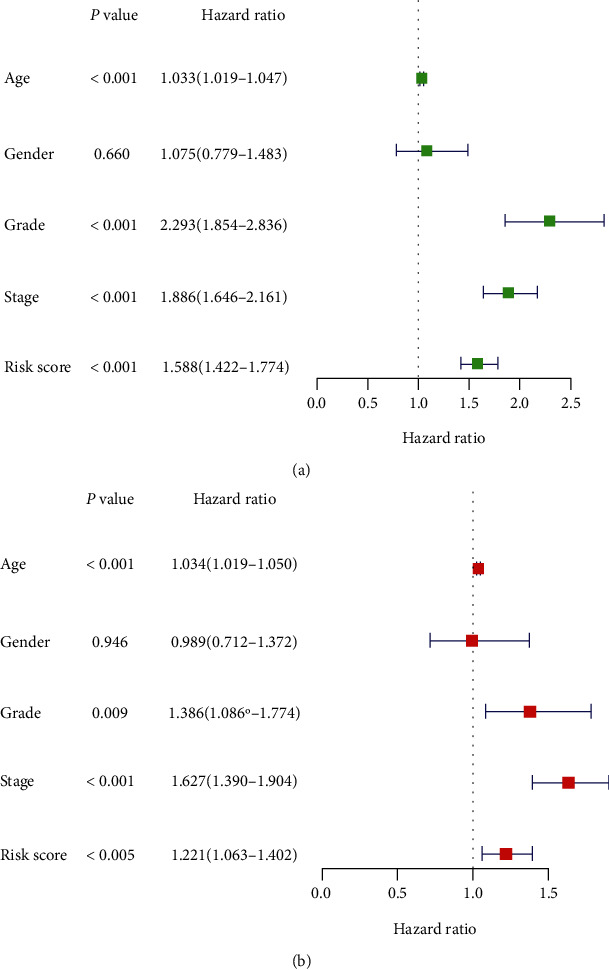
Univariate (a) and multivariate (b) Cox regression analyses of the entire TCGA cohort.

We conducted the univariate and multivariate Cox regression analyses to determine whether the risk score was a prognostic factor of ccRCC independent from traditional clinical features (including age, sex, ISUP grade, and AJCC stage). The results revealed that the risk score was independent of conventional clinical features in the entire TCGA cohort (Figure 6), with a hazard ratio (HR) of 1.221 (95% confidence interval: 1.063–1.402; *P* = 0.005).

**Figure 7:**
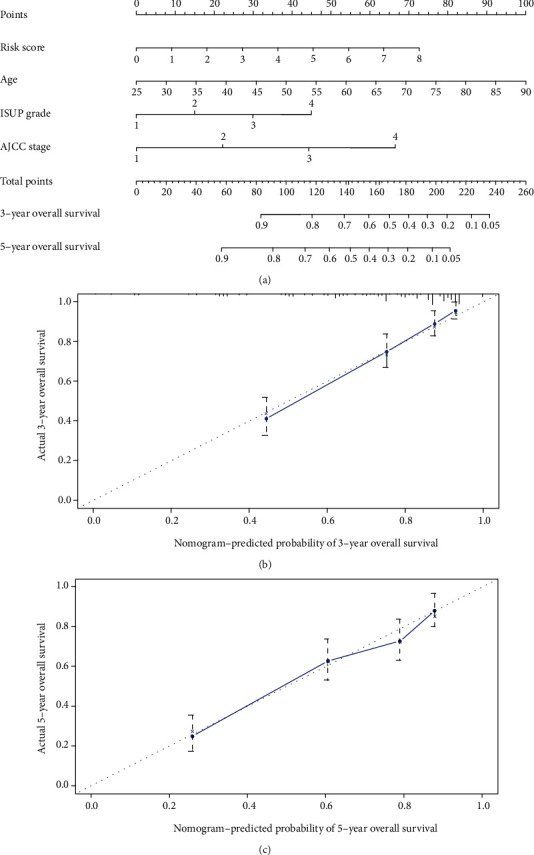
(a) Nomogram predicting 3- and 5-year overall survival for patients with ccRCC. The calibration curve for predicting patient survival at (b) 3 years and (c) 5 years in TCGA datasets. Nomogram-predicted probability of overall survival is plotted on the *x*-axis; actual overall survival is plotted on the *y*-axis.

Then, a nomogram predicting the overall survival in ccRCC was constructed based on the risk score and clinical characteristics (Figure 7(a)). The *C* index was 0.776. The calibration curve showed that the nomogram performed well (Figures 7(b) and 7(c)). Both the *C* index and the calibration curve suggested a good predictive performance.

### 3.6. MethylMix Model of Three Selected DNA MDG

**Figure 8:**
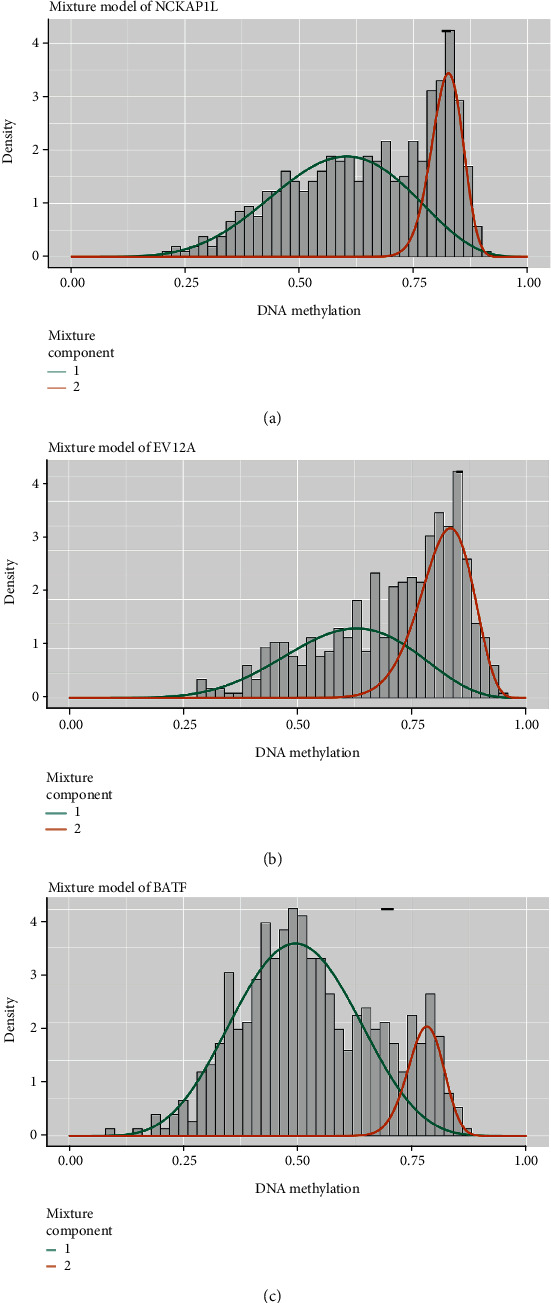
MethylMix model of three selected DNA methylation-driven genes. The distribution maps show the methylation states of methylated genes. The histogram represents the distribution of methylation in tumor samples. The horizontal black bar demonstrates the distribution of methylation in the normal samples. The distribution of the methylation degree can be clearly seen from (a–c).

**Figure 9:**
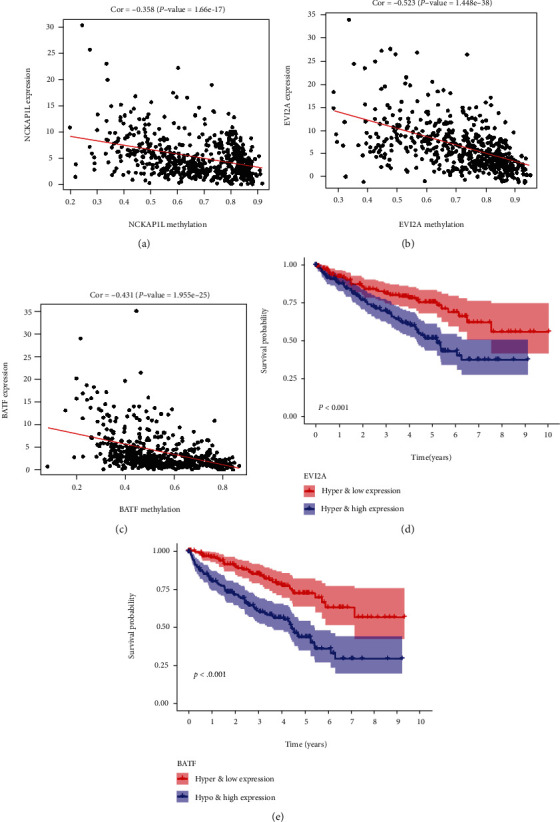
Correlation between DNA methylation and gene expression (a–c) and Kaplan-Meier survival curves for the integrative analysis of EVI2A and BATF (d, e).

The MethylMix model of the three selected DNA MDG is shown in Figure 8. The correlation between DNA methylation and gene expression is visualized in Figures 9(a)–9(c), Cor < −0.3.

### 3.7. Joint Prognostic Assessment of MDG in ccRCC

To further identify the key genes related to the prognosis of ccRCC patients, a joint survival analysis was conducted by combining the methylation levels of the MDG with the corresponding gene expression levels. The results are shown in Figures 9(d) and 9(e). The gene expression and methylation levels of the prognostic genes EVI2A and BATF were significantly related to prognosis.

## 4. Discussion

ccRCC is the most common pathological type of kidney cancer [26]. In-depth molecular pathogenesis research and the early detection of tumor prognostic biomarkers and specific driven-genes may have great significance for improving the prognosis of patients [27–29]. In recent years, with the deepening of epigenetic research, the epigenetic regulatory mechanisms of renal cancer, especially DNA methylation, are often used to predict the survival time of renal cancer, including DAB2IP [30], RCVRN [31], CRHBP [32], AR [33], and CDO1 [34]. However, the heterogeneity of ccRCC limits single-gene methylation in predicting ccRCC outcomes, so it is important to establish a multigene prediction model.

Related studies have shown that abnormal DNA MDG may cause transcriptional disorders, causing some gene expression mistakes and cell differentiation mistakes [35], and tumor suppressor genes and DNA repair genes were silenced due to hypermethylation. DNA methylation alterations at precancerous stages may determine tumor aggressiveness and patient prognosis [36]. Therefore, identifying abnormal DNA MDG can provide new insights for the risk assessment and prognosis of patients.

In this study, we identified key ccRCC-related MDG and constructed a prognostic model based on the level of DNA methylation. First, we screened nineteen methylation-driven genes by analyzing methylation and transcriptome data. Then, to discover the main biological characteristics of these methylation-driven genes, a series of gene functional enrichment analyses were performed. The gene functional enrichment analyses revealed that MDG were significantly linked to inflammatory response regulation and immune regulation (*P* < 0.05, Figure 2).

In addition, a risk score-based prognostic model was constructed for these methylation-driven genes, and the prediction ability of this model was validated in the testing dataset. Three methylation-driven genes (NCKAP1L, EVI2A, and BATF) were identified and used to construct a prognostic risk model for ccRCC. We performed the univariate and multivariate Cox regression analyses on the entire TCGA cohort to verify that the risk model was a prognostic factor for ccRCC independent from other clinicopathological data (age, sex, ISUP grade, and AJCC stage) (HR = 1.221, 95% confidence interval: 1.063–1.402, and *P* = 0.005). In addition, we constructed a nomogram to predict overall survival in patients with ccRCC. The *C* index and calibration curve indicated that the predictive performance of the nomogram was good. Finally, we performed joint survival analysis by combining the methylation levels of the methylation-driven genes with the corresponding gene expression levels. The prognostic genes EVI2A and BATF were significantly related to prognosis.

The gene EVI2A, as the human homolog of mouse genes, may be associated with other proteins in the membrane as a part of a cell surface receptor complex [37]; EVI2A has been shown to be an oncogene [38]; EVI2A is highly expressed in oral tongue squamous cell carcinoma [39] and osteosarcoma [40] and is a risk factor for cancer prognosis. In this study, we found that EVI2A is one of the MDG of ccRCC, and its hypomethylation leads to poor prognosis (Figure 9(d)).

The BATF gene acts a pivotal part in the development of different types of cancer, including colon cancer, lymphoma, and multiple myeloma [41–43]. In addition, Feng et al. found that BATF acted as an oncogene in non-small-cell lung cancer [44]. In this study, the joint survival analyses revealed that the BATF gene was closely related with poor prognosis in patients with ccRCC (Figure 9(e)).

There are some limitations in this study. The specific mechanisms of EVI2A and BATF in ccRCC were not investigated in the previous literature. Therefore, future studies may focus on the molecular mechanisms underlying the potential interactions of EVI2A and BATF in ccRCC.

## 5. Conclusion

In conclusion, we identified key ccRCC-related MDG and constructed a prognostic model based on the level of DNA methylation. In addition, we verified that the risk model was an independent prognostic factor for overall survival in ccRCC. Although further experimental verification is needed, this study provided an important bioinformatics foundation for in-depth studies of ccRCC DNA methylation.

## Figures and Tables

**Table 1:** DNA methylation-driven genes in ccRCC.

Gene	Normal me	Tumor me	logFC	*P* value	Cor	Cor *P* value
C1orf116	0.647716	0.896463	0.468882	3.76*E*-109	-0.6038	4.66*E*-54
ALDOC	0.315874	0.201405	-0.64925	1.32*E*-72	-0.44678	2.04*E*-27
NCKAP1L	0.822054	0.656117	-0.32528	1.06*E*-64	-0.35805	1.66*E*-17
EVI2A	0.854583	0.71327	-0.26077	1.95*E*-62	-0.5227	1.45*E*-38
CD2	0.74397	0.633319	-0.23231	1.57*E*-54	-0.46794	3.01*E*-30
BATF	0.696094	0.535991	-0.37707	3.59*E*-48	-0.43104	1.96*E*-25
CD96	0.703644	0.600698	-0.22821	2.19*E*-45	-0.35773	1.79*E*-17
RAB25	0.498379	0.687697	0.46453	2.40*E*-38	-0.38485	3.44*E*-20
IL20RB	0.886919	0.731896	-0.27716	1.13*E*-33	-0.50225	2.79*E*-35
TMEM173	0.514377	0.327888	-0.64963	4.07*E*-30	-0.36018	1.04*E*-17
CTSZ	0.478134	0.400661	-0.25503	7.68*E*-30	-0.32922	6.89*E*-15
MASP1	0.600411	0.503547	-0.25382	8.74*E*-26	-0.40218	4.61*E*-22
LAMA4	0.426073	0.346439	-0.2985	4.99*E*-22	-0.36743	2.04*E*-18
LTA	0.859977	0.795969	-0.11159	1.31*E*-18	-0.56031	3.21*E*-45
SLC9B2	0.43967	0.392664	-0.16312	3.45*E*-13	-0.38856	1.40*E*-20
FAM107A	0.465871	0.414634	-0.16809	1.77*E*-11	-0.32415	1.86*E*-14
SLC5A12	0.705801	0.634831	-0.15289	1.16*E*-08	-0.33355	2.90*E*-15
AL592211.	0.44045	0.575433	0.38567	0.002048	-0.32193	2.87*E*-14
LRMP	0.534103	0.516217	-0.04914	0.039959	-0.33687	1.48*E*-15

## Data Availability

The data included in the current study was available in TCGA database (https://cancergenome.nih.gov/).

## Supplementary Materials

Supplementary Materials Table S1 a training cohort. Table S2 a validation cohort. Table S3 normal DNA methylation data. Table S4 cancer DNA methylation data. Table S5 matched gene expression data.

## References

[1] Jonasch E., Gao J., Rathmell W. K. (2014). Renal cell carcinoma. *BMJ*.

[2] Shuch B., Amin A., Armstrong A. J. (2015). Understanding pathologic variants of renal cell carcinoma: distilling therapeutic opportunities from biologic complexity. *European Urology*.

[3] Capitanio U., Montorsi F. (2016). Renal cancer. *The Lancet*.

[4] MacLennan S., Imamura M., Lapitan M. C. (2012). Systematic review of oncological outcomes following surgical management of localised renal cancer. *European Urology*.

[5] Bazzi W. M., Sjoberg D. D., Feuerstein M. A. (2015). Long-term survival rates after resection for locally advanced kidney cancer: Memorial Sloan Kettering Cancer Center 1989 to 2012 experience. *The Journal of Urology*.

[6] Teng J., Gao Y., Chen M. (2014). Prognostic value of clinical and pathological factors for surgically treated localized clear cell renal cell carcinoma. *Chinese Medical Journal*.

[7] Miller K. D., Nogueira L., Mariotto A. B. (2019). Cancer treatment and survivorship statistics, 2019. *CA: A Cancer Journal for Clinicians*.

[8] Shen C., Kaelin W. G. (2013). The VHL/HIF axis in clear cell renal carcinoma. *Seminars in Cancer Biology*.

[9] Wang Z., Peng S., Guo L. (2018). Prognostic and clinicopathological value of PBRM1 expression in renal cell carcinoma. *Clinica Chimica Acta*.

[10] Atkins M. B., Tannir N. M. (2018). Current and emerging therapies for first-line treatment of metastatic clear cell renal cell carcinoma. *Cancer Treatment Reviews*.

[11] Zheng X., Zhang N., Wu H. J., Wu H. (2017). Estimating and accounting for tumor purity in the analysis of DNA methylation data from cancer studies. *Genome Biology*.

[12] Bernstein B. E., Meissner A., Lander E. S. (2007). The mammalian epigenome. *Cell*.

[13] Pogribny I. P., Beland F. A. (2009). DNA hypomethylation in the origin and pathogenesis of human diseases. *Cellular and Molecular Life Sciences*.

[14] Wang C., Zhao N., Yuan L., Liu X. (2020). Computational detection of breast cancer invasiveness with DNA methylation biomarkers. *Cell*.

[15] Wang Z., Yin J., Zhou W. (2020). Complex impact of DNA methylation on transcriptional dysregulation across 22 human cancer types. *Nucleic Acids Research*.

[16] El‐Zein M., Cheishvili D., Gotlieb W. (2020). Genome-wide DNA methylation profiling identifies two novel genes in cervical neoplasia. *International Journal of Cancer*.

[17] Feng H., Zhang Z., Qing X., Wang X., Liang C., Liu D. (2016). Promoter methylation of APC and RAR-*β* genes as prognostic markers in non-small cell lung cancer (NSCLC). *Experimental and Molecular Pathology*.

[18] Zhang S., Wang Y., Gu Y. (2018). Specific breast cancer prognosis-subtype distinctions based on DNA methylation patterns. *Molecular Oncology*.

[19] Hao X., Luo H., Krawczyk M. (2017). DNA methylation markers for diagnosis and prognosis of common cancers. *Proceedings of the National Academy of Sciences of the United States of America*.

[20] Zhao J., Le Wang D. K., Hu G., Wei B. (2020). Construction of novel DNA methylation-based prognostic model to predict survival in glioblastoma. *Journal of Computational Biology*.

[21] Ritchie M. E., Phipson B., Wu D. (2015). Limma powers differential expression analyses for RNA-sequencing and microarray studies. *Nucleic Acids Research*.

[22] Gevaert O. (2015). MethylMix: an R package for identifying DNA methylation-driven genes. *Bioinformatics (Oxford, England)*.

[23] Gevaert O., Tibshirani R., Plevritis S. K. (2015). Pancancer analysis of DNA methylation-driven genes using MethylMix. *Genome Biology*.

[24] Gene Ontology Consortium (2006). The Gene Ontology (GO) project in 2006. *Nucleic Acids Research*.

[25] Yu G., Wang L. G., Han Y., He Q. Y. (2012). clusterProfiler: an R package for comparing biological themes among gene clusters. *OMICS*.

[26] Chen F., Zhang Y., Senbabaoglu Y. (2016). Multilevel genomics-based taxonomy of renal cell carcinoma. *Cell Reports*.

[27] Li R., Yang Y. E., Yin Y. H., Zhang M. Y., Li H., Qu Y. Q. (2019). Methylation and transcriptome analysis reveal lung adenocarcinoma-specific diagnostic biomarkers. *Journal of Translational Medicine*.

[28] Kanai Y. (2010). Genome-wide DNA methylation profiles in precancerous conditions and cancers. *Cancer Science*.

[29] Arai E., Kanai Y. (2010). Genetic and epigenetic alterations during renal carcinogenesis. *International Journal of Clinical and Experimental Pathology*.

[30] Wang Z. R., Wei J. H., Zhou J. C. (2016). Validation of DAB2IP methylation and its relative significance in predicting outcome in renal cell carcinoma. *Oncotarget*.

[31] Golovastova M. O., Tsoy L. V., Bocharnikova A. V. (2016). The cancer-retina antigen recoverin as a potential biomarker for renal tumors. *Tumour Biology*.

[32] Tezval H., Dubrowinskaja N., Peters I. (2016). Tumor specific epigenetic silencing of corticotropin releasing hormone -binding protein in renal cell carcinoma: association of hypermethylation and metastasis. *PLoS One*.

[33] Zhao H., Leppert J. T., Peehl D. M. (2016). A protective role for androgen receptor in clear cell renal cell carcinoma based on mining TCGA data. *PLoS One*.

[34] Deckers I. A. G., Schouten L. J., Van Neste L. (2015). Promoter methylation of CDO1 identifies clear-cell renal cell cancer patients with poor survival outcome. *Clinical Cancer Research*.

[35] Morris M. R., Ricketts C. J., Gentle D. (2011). Genome-wide methylation analysis identifies epigenetically inactivated candidate tumour suppressor genes in renal cell carcinoma. *Oncogene*.

[36] Arai E., Chiku S., Mori T. (2012). Single-CpG-resolution methylome analysis identifies clinicopathologically aggressive CpG island methylator phenotype clear cell renal cell carcinomas. *Carcinogenesis*.

[37] Cawthon R. M., O'Connell P., Buchberg A. M. (1990). Identification and characterization of transcripts from the neurofibromatosis 1 region: the sequence and genomic structure of EVI2 and mapping of other transcripts. *Genomics*.

[38] Cawthon R. M., Andersen L. B., Buchberg A. M. (1991). cDNA sequence and genomic structure of EVI2B, a gene lying within an intron of the neurofibromatosis type 1 gene. *Genomics*.

[39] Qiu Z., Sun W., Gao S. (2017). A 16-gene signature predicting prognosis of patients with oral tongue squamous cell carcinoma. *PeerJ*.

[40] Li S., Yang F., Yang Y.-K., Zhou Y. (2019). Increased expression of ecotropic viral integration site 2A indicates a poor prognosis and promotes osteosarcoma evolution through activating MEK/ERK pathway. *Journal of Receptor and Signal Transduction Research*.

[41] Dai L., Cui X., Zhang X. (2016). SARI inhibits angiogenesis and tumour growth of human colon cancer through directly targeting ceruloplasmin. *Nature Communications*.

[42] Schleussner N., Merkel O., Costanza M. (2018). The AP-1-BATF and -BATF3 module is essential for growth, survival and TH17/ILC3 skewing of anaplastic large cell lymphoma. *Leukemia*.

[43] Gil M., Pak H. K., Park S. J. (2015). Engagement of CD99 reduces AP-1 activity by inducing BATF in the human multiple myeloma cell line RPMI8226. *Immune Network*.

[44] Feng Y., Pan L., Zhang B., Huang H., Ma H. (2020). BATF acts as an oncogene in non-small cell lung cancer. *Oncology Letters*.

